# Arabidopsis mutants impaired in glutathione biosynthesis exhibit higher sensitivity towards the glucosinolate hydrolysis product allyl-isothiocyanate

**DOI:** 10.1038/s41598-018-28099-1

**Published:** 2018-06-28

**Authors:** János Urbancsok, Atle M. Bones, Ralph Kissen

**Affiliations:** Cell, Molecular Biology and Genomics Group, Department of Biology, Norwegian University of Science and Technology, NO-7491 Trondheim, Norway

## Abstract

Upon tissue damage the plant secondary metabolites glucosinolates can generate various hydrolysis products, including isothiocyanates (ITCs). Their role in plant defence against insects and pest and their potential health benefits have been well documented, but our knowledge regarding the endogenous molecular mechanisms of their effect in plants is limited. Here we investigated the effect of allyl-isothiocyanate (AITC) on *Arabidopsis thaliana* mutants impaired in homeostasis of the low-molecular weight thiol glutathione. We show that glutathione is important for the AITC-induced physiological responses, since mutants deficient in glutathione biosynthesis displayed a lower biomass and higher root growth inhibition than WT seedlings. These mutants were also more susceptible than WT to another ITC, sulforaphane. Sulforaphane was however more potent in inhibiting root growth than AITC. Combining AITC with the glutathione biosynthesis inhibitor L-buthionine-sulfoximine (BSO) led to an even stronger phenotype than observed for the single treatments. Furthermore, transgenic plants expressing the redox-sensitive fluorescent biomarker roGFP2 indicated more oxidative conditions during AITC treatment. Taken together, we provide genetic evidence that glutathione plays an important role in AITC-induced growth inhibition, although further studies need to be conducted to reveal the underlying mechanisms.

## Introduction

Glucosinolates (GSLs) are secondary metabolites produced by species belonging to the Brassicales order, hence are present in the model plant *Arabidopsis thaliana*. GSLs are hydrolysed by enzymes called myrosinases (β-thioglucosidases) when they come together upon tissue damage^1,2^. Due to this enzymatic reaction, a variety of glucosinolate hydrolysis products (GHPs) can be generated such as isothiocyanates (ITCs), nitriles, epithionitriles and thiocyanates depending on the GSL substrate and reaction conditions^3^. ITCs are the most commonly generated breakdown products and are considered as biologically active phytochemicals in plant-insect and plant-pathogen interactions^4,5^. Different ITCs have also been involved in the inhibition of carcinogenesis^6–8^.

Allyl-isothiocyanate (AITC) originates from the enzymatic cleavage of the aliphatic GSL sinigrin and has been shown to exhibit antibacterial, antifungal and antinematodal properties^9–11^. In addition, AITC affects the behaviour, growth and survival of insects^12,13^. A growing body of scientific evidence on mammalian systems indicates that AITC treatment arrests the cell cycle, causes apoptosis and inhibits the proliferation of cancer cells leading to reduced tumour formation in certain types of cancer^14,15^. Our understanding is however relatively poor about the biological effects of AITC and other isothiocyanates on plants. AITC was studied as a possible alternative to hazardous chemicals in weed control management since it can act as herbicide at high concentrations^16,17^. Exogenously applied AITC causes stomatal closure via the generation of reactive oxygen species (ROS) and enhances the heat tolerance in *A. thaliana*^18–20^. Our group has lately demonstrated that AITC causes a shift in the cell cycle distribution, leads to a disintegration of microtubules and inhibition of the actin-dependent intracellular transport in *A. thaliana*^21–23^.

ITCs are electrophilic compounds that have been shown to activate phase II detoxification enzymes, including glutathione *S*-transferases (GSTs), in several organisms^24–26^. Some GSTs are capable of mediating the conjugation of the available sulfhydryl group of reduced glutathione (GSH) to the electrophilic central carbon atom of the isothiocyanate group^27–30^. The conjugation of ITCs to glutathione can however also be non-enzymatic^29,31,32^. In mammals this chemical interaction results in the formation of mercapturic acid through enzymatic or non-enzymatic steps followed by excretion^33,34^. Compared to animal systems, there is limited knowledge about GST-mediated *in vivo* conjugation of AITC to glutathione for plants^35,36^. But some plant GSTs show activity on ITCs *in vitro*^37–39^, and exogenous application of AITC upregulates transcript levels of *GST*s and depletes glutathione^17,40,41^.

**Figure 1:**
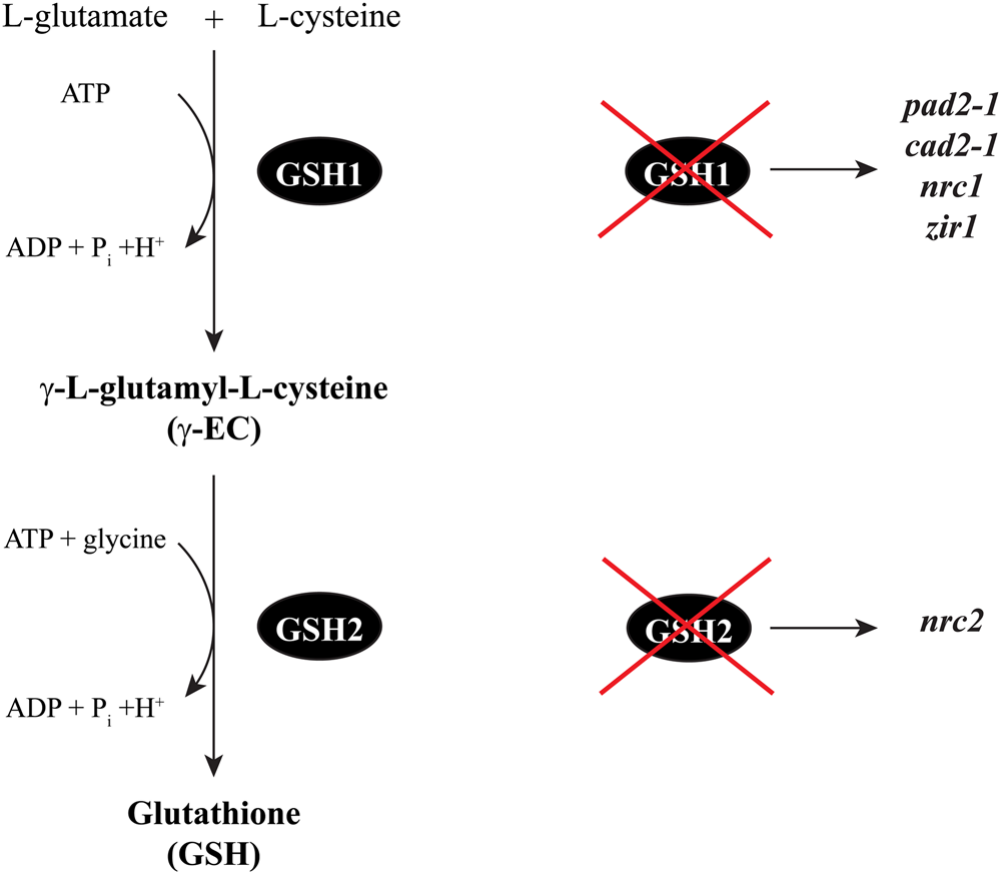
Glutathione biosynthesis and mutants. On the left, schematic representation of the glutathione biosynthetic pathway. On the right, simplified explanatory graph showing which gene of GSH biosynthesis is impaired in the particular mutants.

Glutathione is synthesized in a two-step process, in which the first and rate-limiting enzyme γ-glutamylcysteine synthetase (γ–ECS or GSH1) converts glutamate and cysteine to γ–EC, then glutathione synthetase (GSH2) forms glutathione by the ligation of an additional glycine to the intermediate^42,43^ (Fig. 1). In plants glutathione plays a pivotal role in various physiological responses such as redox homeostasis and ROS scavenging, detoxification of xenobiotics and development^44–46^. Glutathione is also involved in the biosynthesis of phytochelatins and defence compounds such as camalexin and glucosinolates^47–49^.

Nowadays, the importance of GHPs has gained significant attention, especially understanding their potential effects on plants. In the present study we investigated the role of glutathione in the response of *A. thaliana* to AITC in order to better understand the underlying molecular mechanisms of ITC-induced physiological responses. We show that *A. thaliana GSH1* and *GSH2* mutants, affected in the biosynthesis of glutathione, have reduced root growth and total biomass production compared to wild type (WT) seedlings when exposed to exogenous AITC. A series of mutants impaired in other genes putatively involved in glutathione homeostasis were however not more susceptible than WT. Our results show that the low-molecular weight thiol glutathione plays an essential role in ITC-triggered physiological responses.

## Results

### Glutathione-deficient mutants showed an increased susceptibility to AITC

**Figure 2:**
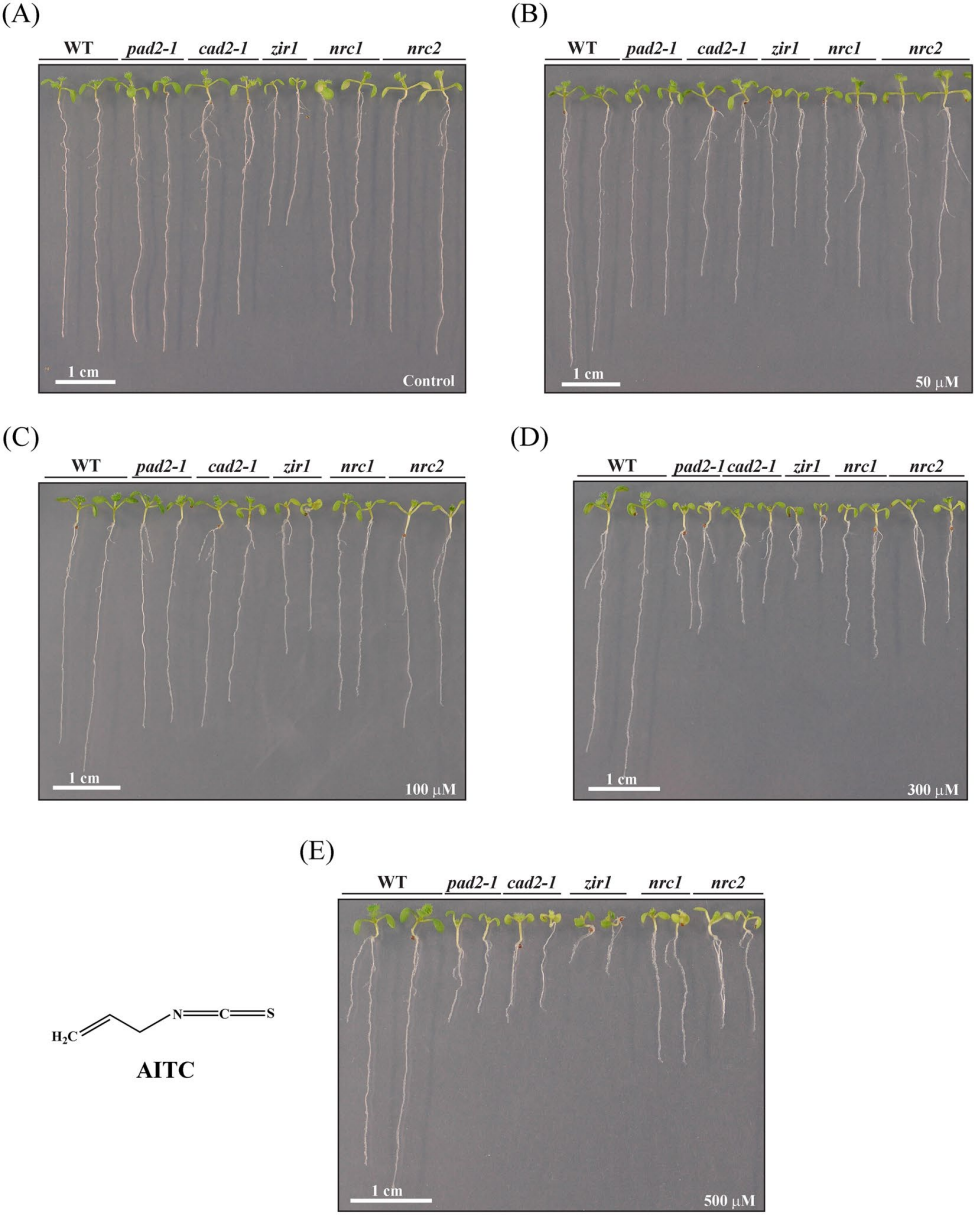
Effect of AITC on seedling growth. Ten day old WT and glutathione mutants grown in a vertical position on solid *in vitro* medium supplemented with AITC at the desired concentrations are shown: (**A**) control, (**B**) 50 μM, (**C**) 100 μM, (**D**) 300 μM and (**E**) 500 μM. The structure of AITC is also shown. For illustration purposes two plants of each line from plates at the same concentration were transferred together to a separate plate on day 10.

**Figure 3:**
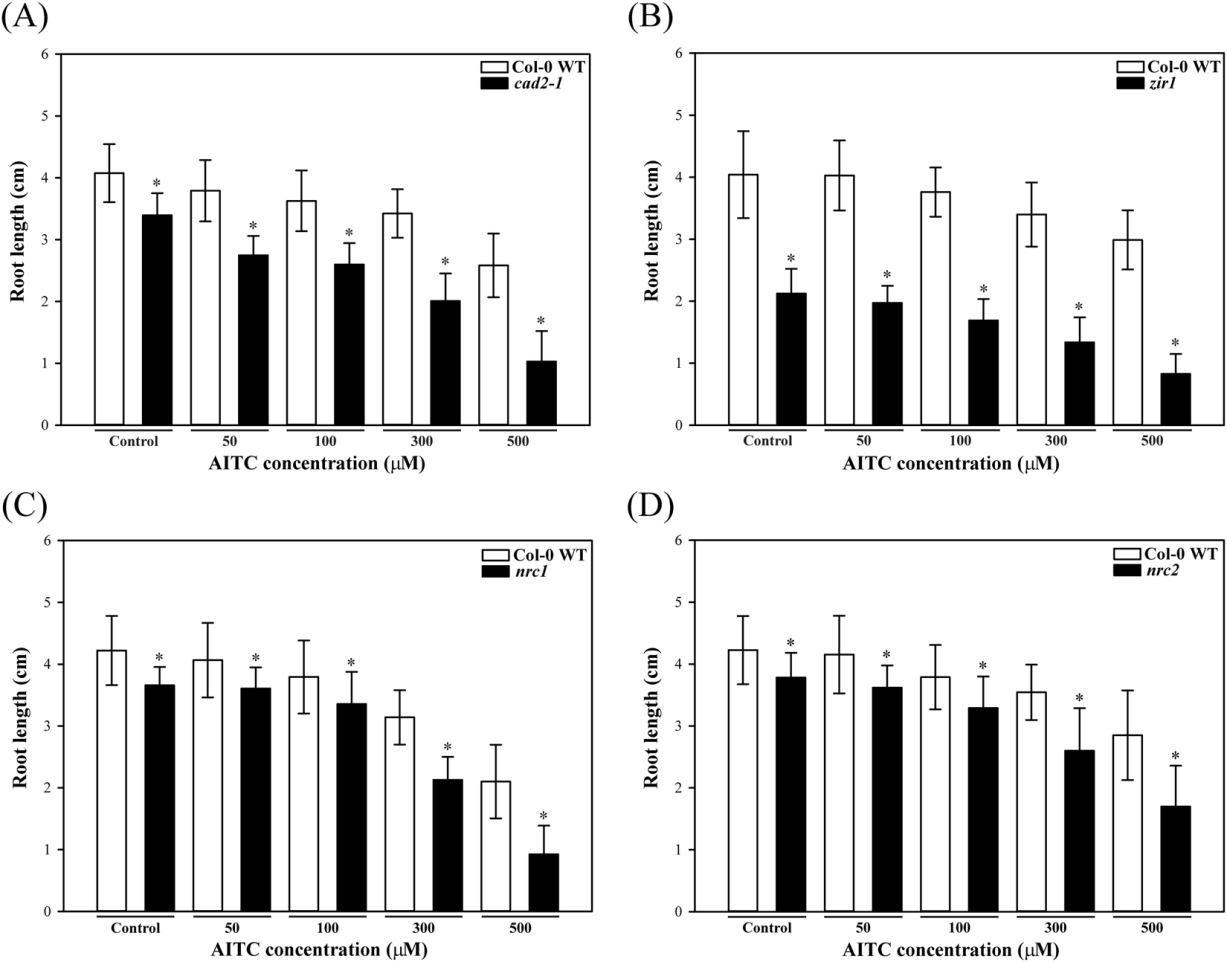
Root growth inhibition by AITC. Mean root length (n = 60) of 10 day old seedlings of the WT and the glutathione mutants (**A**) *cad2-1*, (**B**) *zir1*, (**C**) *nrc1* and (**D**) *nrc2* grown on solid *in vitro* medium supplemented with different concentrations of AITC. Asterisks indicate that the difference is significant between the WT and the particular mutant (P < 0.01, Student’s t-test) for that treatment. Error bars represent SDs.

To investigate the importance of glutathione in the ITC-triggered response of *A. thaliana* the five mutants *pad2-1*, *cad2-1*, *zir1*, *nrc1* and *nrc2* that are affected in glutathione biosynthesis as they are either impaired in GSH1 or GSH2 (Fig. 1; Supplementary Table S1) and Col-0 WT seedlings were exposed to different concentrations of AITC. Under the control treatment all the mutants, especially *zir1*, *nrc1* and *nrc2*, showed reduced growth compared to the WT (Fig. 2A), which is consistent with some earlier observations^50,51^. The AITC treatment led to a dose-dependent growth inhibition of Col-0 WT seedlings as shown in Fig. 2 and measured by primary root length on day 10 (Fig. 3).

Mutants that are impaired in glutathione biosynthesis exhibited a stronger growth inhibition than WT seedlings when exposed to AITC, independent of whether the mutation was present in the first or second enzyme of glutathione biosynthesis (Figs 2 and 3). The AITC-induced root growth reduction was greater for the glutathione mutants than the WT, especially at higher doses, with the largest AITC inhibition effect observed for *nrc1* and *cad2-1*. To account for the fact that glutathione mutants had shorter roots on the control plates, ratios between the average root length on each AITC concentration and on control medium are shown in Supplementary Figure S1. Although *zir1* had much shorter roots on control medium and exhibited the strongest phenotype on AITC (Fig. 2), the ratio to the control treatment showed that the root growth of *zir1* was affected similarly to that of the other glutathione mutants (Supplementary Figure S1).

We supplemented the growth medium with glutathione monoethyl ester in addition to AITC but this did not mitigate the AITC-induced root growth inhibition of WT or mutant seedlings (data not shown).

### Dynamics of the AITC-induced effect on root elongation in glutathione mutants

To assess the dynamics of the differential AITC effect on root growth between glutathione mutants and WT seedlings the root elongation on 300 µM AITC was monitored at different time points. The results clearly indicated that the five glutathione mutants had shorter roots on control plates than the WT already after 4 days (Supplementary Figure S2). At this time point the reduction of root growth by AITC was substantially smaller for *zir1* than for the other lines. Between day 4 and day 7 the root elongation of all glutathione mutants was remarkably different from that of WT. While AITC affected the root length increment of WT by 20% over this period it had a much stronger effect on the glutathione mutants. From day 7 to day 10 the differences between control and AITC-treated seedlings were smaller than those observed during the second period, indicating that the effect of AITC on root growth persisted until day 10 but decreased over time (Supplementary Figure S2).

### Glutathione mutants produced lower biomass upon AITC treatment

**Figure 4:**
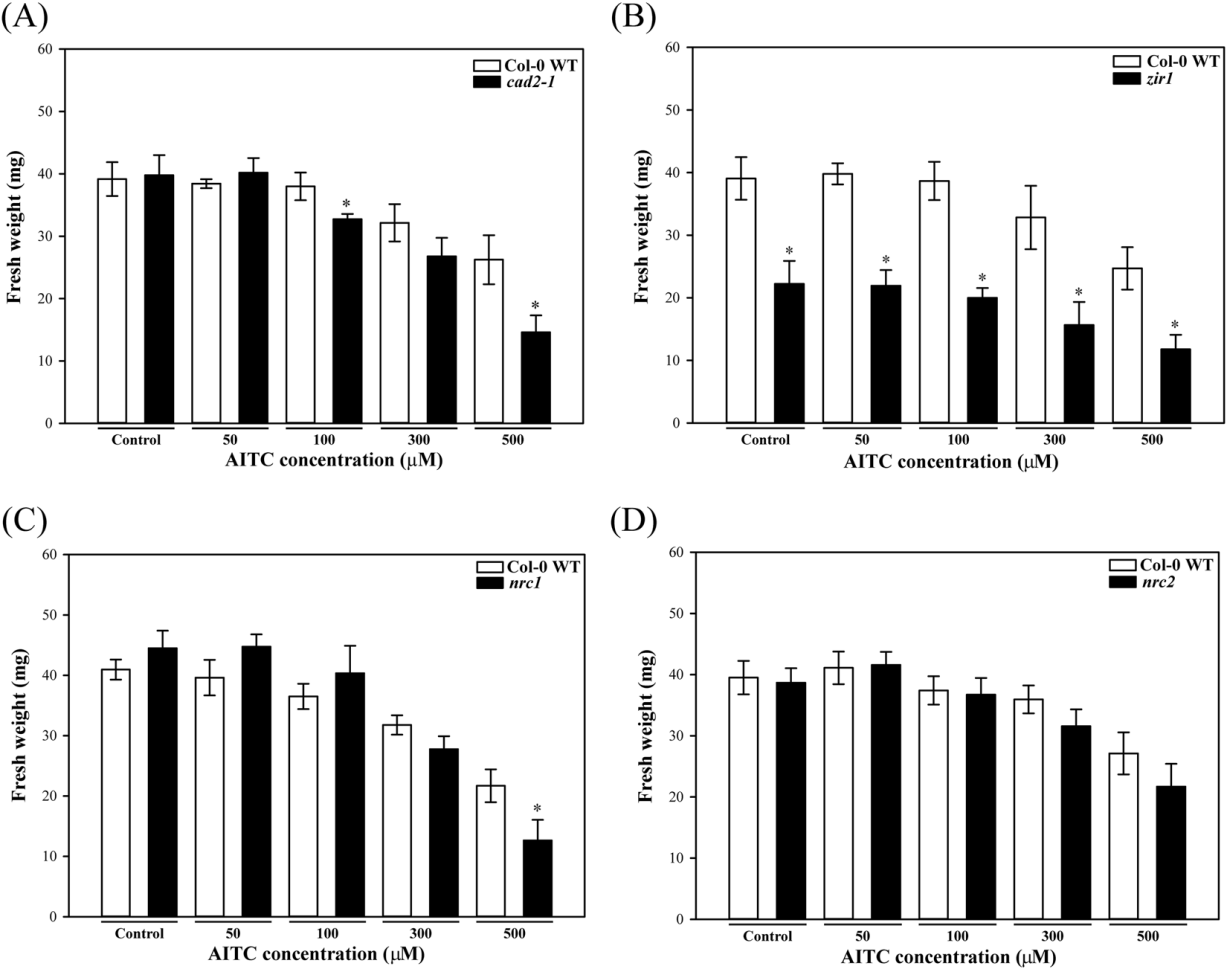
Effect of AITC on seedling biomass. The mean fresh weight of WT and glutathione mutants: (**A**) *cad2-1*, (**B**) *zir1*, (**C**) *nrc1*, (**D**) *nrc2* grown on medium supplemented with different concentrations of AITC was measured on day 10 (n = 4, each replicate consists of a pool of 15 seedlings). Statistically significant differences between the WT and the particular mutant for a given treatment are marked by asterisks (P < 0.01, Student’s t-test). Error bars represent SDs.

The total biomass was also investigated following AITC treatment and showed that in contrast to root length, fewer significant differences in fresh weight values were observed between WT and the glutathione mutants (Fig. 4). On control plates the biomass of glutathione mutants was not different from the WT, except for *zir1* (Fig. 4B). The biomass of glutathione mutants was consistently more inhibited by 300 μM and 500 μM AITC treatments than that of the WT, which is similar to the root length results described above. Despite the slightly higher fresh weight of *nrc1* at low AITC concentrations the mutant coped less well at the two highest doses relative to WT (Fig. 4C and Supplementary Figure S3).

### Glutathione mutants showed also a higher susceptibility to SFN treatment

**Figure 5:**
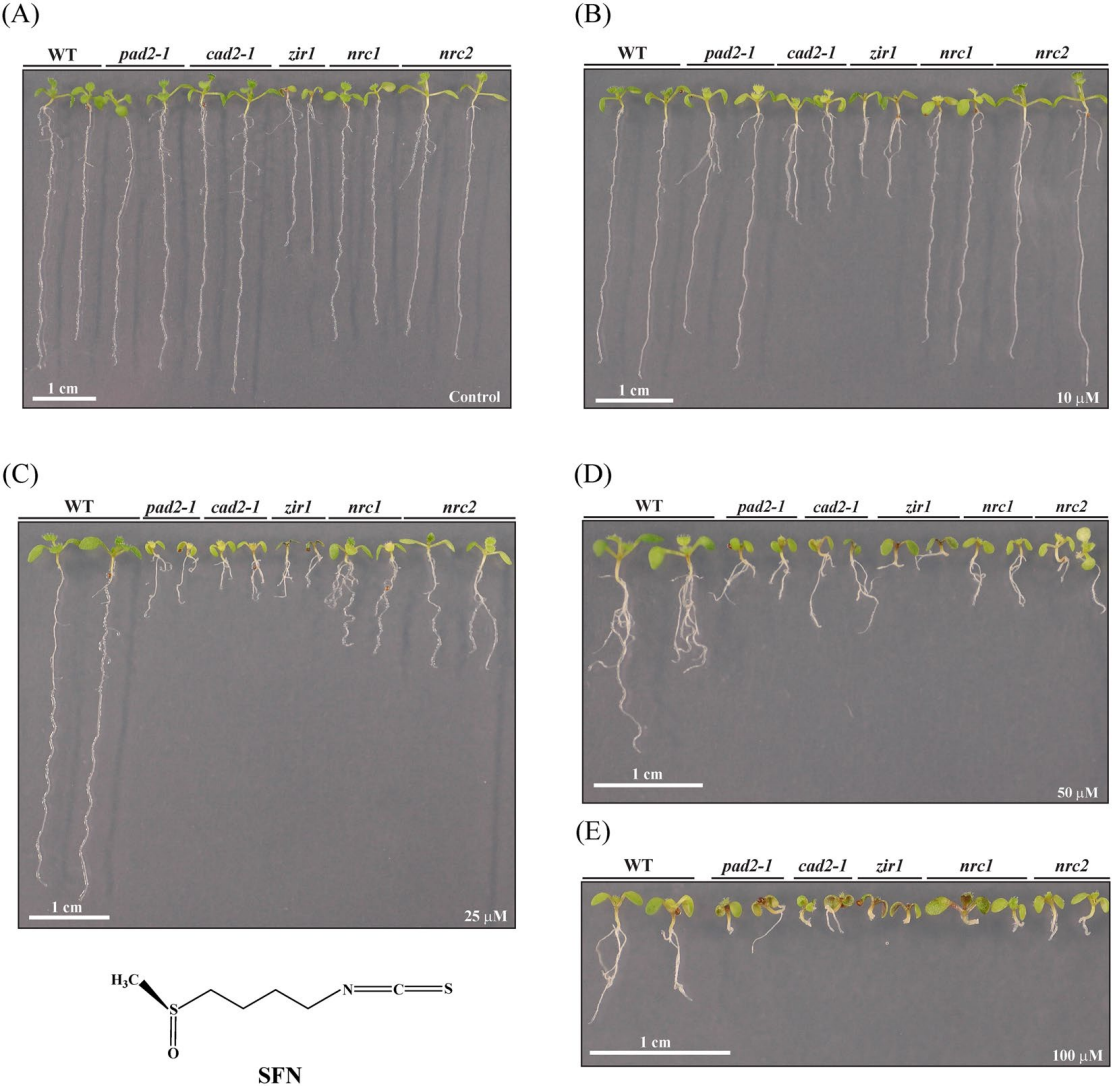
Effect of SFN on seedling growth. Representative 10 day old WT and glutathione mutant seedlings grown on plates supplemented with SFN at the indicated concentrations are shown: (**A**) control, (**B**) 10 μM, (**C**) 25 μM, (**D**) 50 μM and (**E**) 100 μM. The structure of SFN is also shown.

To address the question if the more susceptible phenotype of glutathione mutants towards AITC was unique for this GHP, sulforaphane (SFN), an aliphatic ITC possessing a longer side chain than AITC, was tested. Similar to the exposure to high AITC concentrations the five glutathione mutants were more severely affected by SFN than WT. Mutants such as *cad2-1* and *zir1* exhibited shorter roots already at the lowest SFN dose, and clear differences to the WT were observed for all glutathione mutants on 25 μM and 50 μM SFN (Fig. 5). Furthermore the highest SFN concentration tested (100 µM) led to extreme root growth inhibition (Fig. 5), revealing a much higher potency for SFN than for AITC.

### Transgenic lines expressing roGFP2 indicated a change in the glutathione redox potential upon AITC treatment

**Figure 6:**
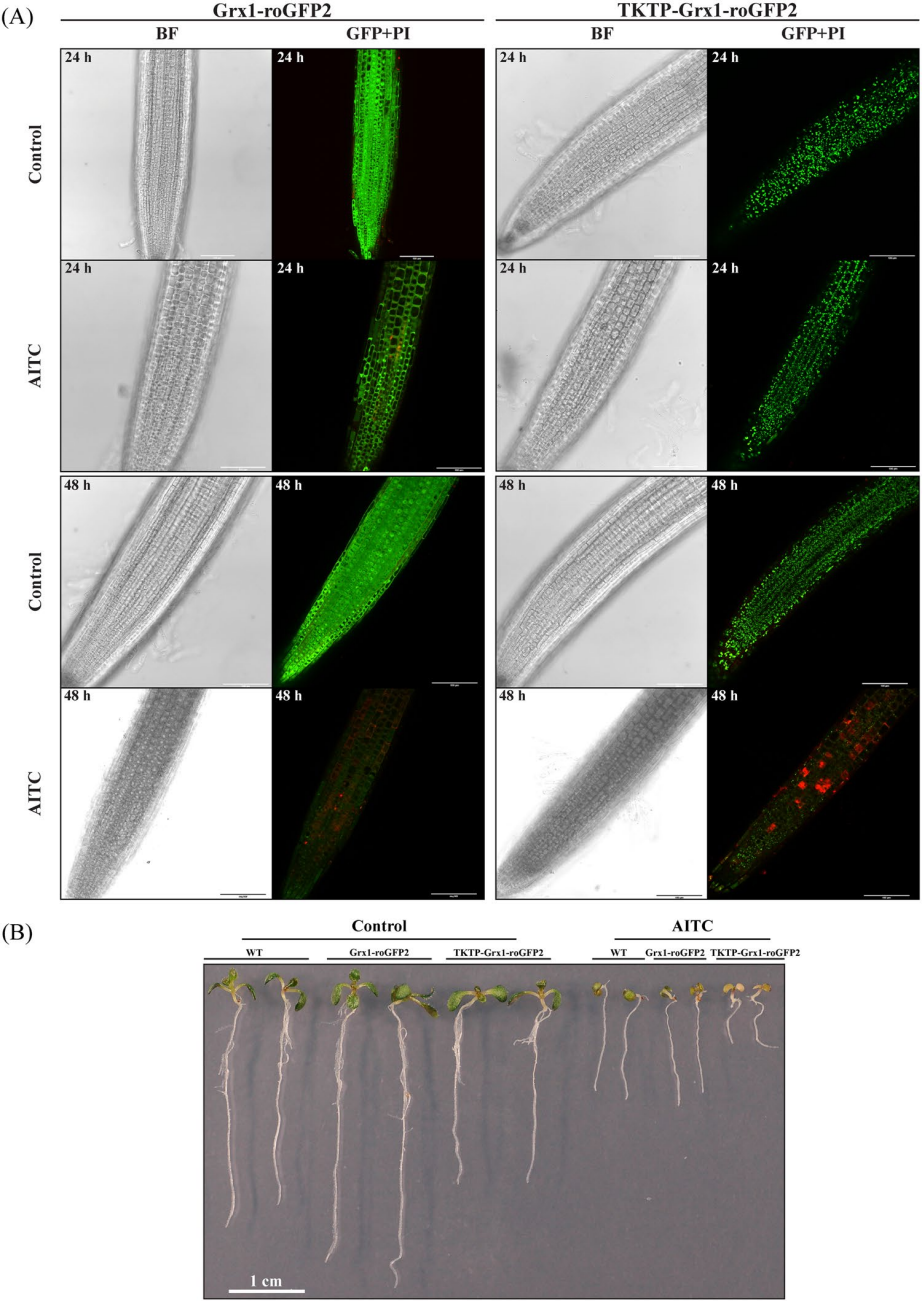
Fluorescence of roGFP2 seedlings at different time points of a 1 mM AITC treatment. (**A**) Representative confocal images (BF: brightfield, GFP + PI: overlay image of GFP excited at 488 nm and propidium iodide fluorescent signals) of roots exposed to AITC for 24 h and 48 h. Intensive red staining in AITC-treated roots indicates cell death. Scale bars = 100 µm. (**B**) Representative pictures of WT and roGFP2 plantlets after 72 h of AITC treatment as under (**A**) demonstrate the AITC-triggered growth phenotype.

The application of AITC led to stronger responses for the glutathione mutants than for WT, which may suggest an essential role for this tripeptide in the AITC-induced physiological responses. In order to elucidate whether the glutathione redox potential was changed or not during the treatment we used two transgenic Arabidopsis lines expressing the versatile redox-sensitive fluorescent protein roGFP2 that is a biosensor for the glutathione redox potential^52^. The cytosolic Grx1-roGFP2 and to a lesser extent the plastidic TKTP-Grx1-roGFP2 lines exhibited lower fluorescence intensity when excited at 488 nm after 24 h or 48 h of AITC treatment compared to their respective control, indicating oxidative conditions and/or lower levels of glutathione (Fig. 6A). In roots of seedlings that were exposed to AITC for 72 h roGFP2 fluorescence was almost completely absent (data not shown). In addition to the intensive propidium iodide fluorescence of root cell nuclei indicating cell death in AITC-treated roots (Fig. 6A), seedlings also exhibited growth inhibition especially following the 72 h treatment (Fig. 6B).

### The combination of L-buthionine-sulfoximine and AITC exacerbated the growth inhibition

L-buthionine-sulfoximine (BSO) is a specific inhibitor of GSH1. Its application to plants leads to depletion of the cellular glutathione pool^53^, the inhibition of root growth^54^ and a growth reduction similar to that observed for the severely glutathione deficient *rml1* (*root meristemless1*)^55^. As AITC treatment led to a similar biological outcome we compared the growth inhibition effects caused by AITC, BSO and a combination of the two chemicals on WT and *pad2-1* seedlings.

**Figure 7:**
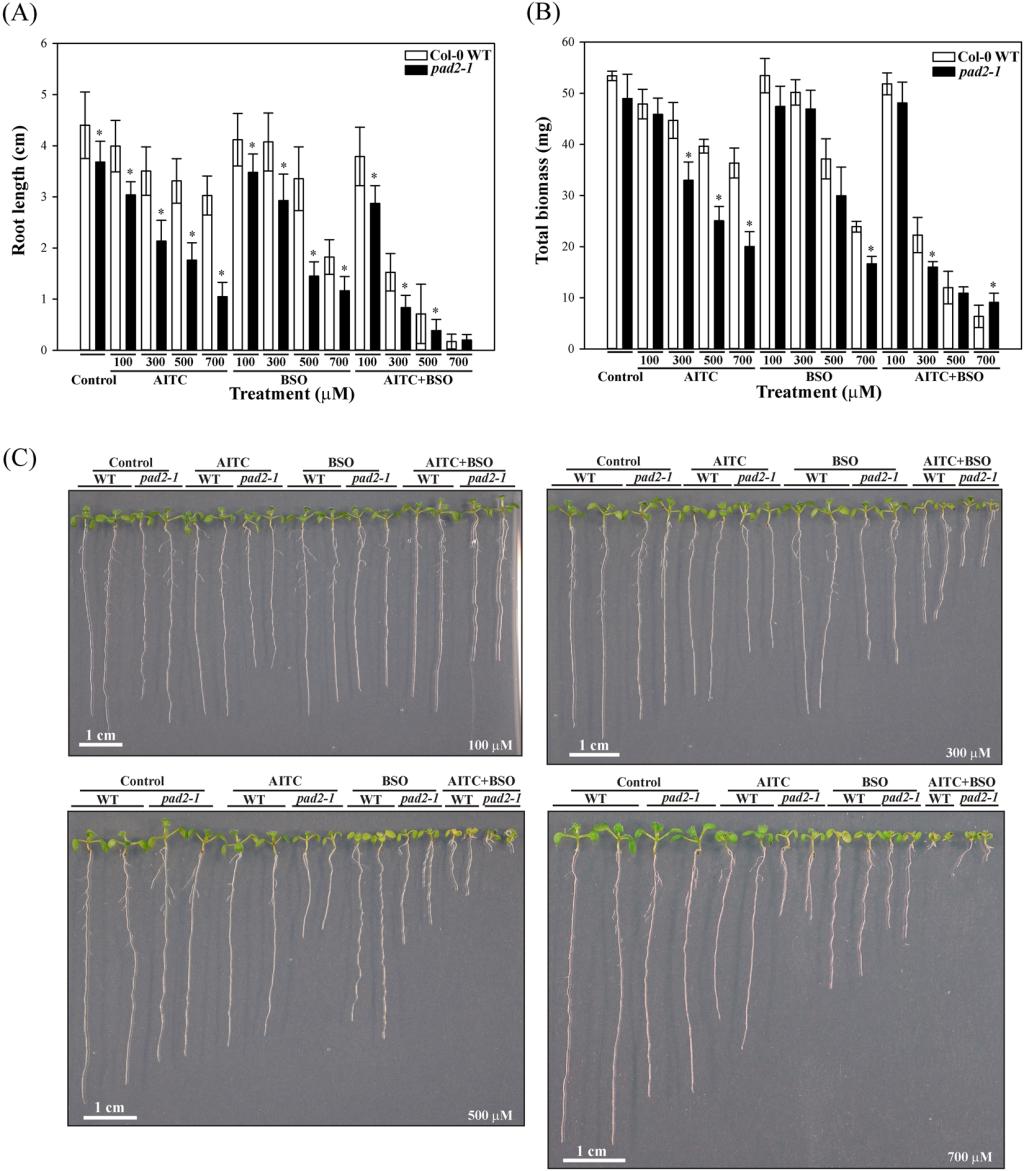
Growth inhibition effects of AITC, BSO and their combination on WT and *pad2-1* seedlings. (**A**) Mean root lengths of 10 day old seedlings (n = 60) grown on solid *in vitro* medium supplemented with the chemicals at the given concentration levels. (**B**) Mean biomass of 10 day old seedlings (n = 4 and each of them consists of a pool of 15 seedlings) grown on plates supplemented with different concentrations of the compounds. (**C**) Ten day old WT and *pad2-1* plants exposed to the chemicals at the indicated concentrations (bottom right corners). In (**A**) and (**B**) error bars represent SDs and asterisks mark a significant difference (P < 0.01, Student’s t-test) between *pad2-1* and WT for a given treatment.

AITC led to reduced root growth, with a more severe reduction for *pad2-1* than for WT (Fig. 7A and Supplementary Figure S4). Col-0 WT seedlings exposed to BSO showed similar root inhibition as when exposed to equivalent amounts of AITC, except at the highest concentration. BSO had similar effects on Col-0 WT and *pad2-1* seedlings at 100 µM whereas it inhibited the root growth of *pad2-1* more than of WT at the three highest BSO concentrations. The growth inhibition effect of BSO on *pad2-1* seemed however to reach a plateau at the highest dose, in contrast to Col-0. The combination of AITC and BSO resulted in more severe root growth inhibition compared to the single treatments (Fig. 7A and C). The effect of the combined treatment was also stronger on *pad2-1* than on WT, except for a concentration of 700 µM each (Fig. 7C and Supplementary Figure S4).

The total biomass was also affected by these treatments, although fewer significant differences were detected (Fig. 7B). The biomass of *pad2-1* was more affected than that of WT by both AITC and BSO. The combined application of AITC and BSO caused more severe effects on fresh weight than the single treatments, consistent with results of the combined treatment on root length. Moreover, *pad2-1* exhibited significantly higher biomass than WT at 700 μM (Fig. 7B and Supplementary Figure S4).

### Mutants affected in the transport and degradation of glutathione and glutathione S-conjugates did not exhibit differences in their susceptibility to AITC

While glutathione is synthesized in the chloroplast and the cytosol it is also present in the mitochondrion, nucleus and the apoplast, indicating the necessity of transport^56^. Oxidized glutathione and glutathione S-conjugates that the low-molecular weight thiol forms with endogenous and xenobiotic electrophilic compounds can also be transported between subcellular compartments and across the plasma membrane. As these transport mechanisms take part in glutathione homeostasis we tested the susceptibility of different transporter mutants (Supplementary Table S1) to AITC. As none of these showed any difference with WT in their susceptibility to AITC the data is not presented in detail. The triple mutant *clt1clt2clt3* is deficient in the transport of γ–EC and glutathione from the plastid to the cytosol and shows lower cytosolic and higher plastidic glutathione levels^57^, but did not exhibit any difference in root length compared to WT when exposed to AITC. Multidrug Resistance-associated Protein 1 (MRP1) and MRP2, of the ABC (ATP-Binding Cassette)-type subfamily C transporters, transport oxidized glutathione and glutathione S-conjugates when expressed in yeast^58,59^ and might be involved in their transport from the cytosol into the vacuole in plants. MRP3 has also been reported to transport glutathione S-conjugates^60^. The root growth of mutants affected in these three *MRPs* was not distinguishable from that of WT upon AITC treatment. Members of the oligopeptide transporters (OPTs) might be responsible for transport of glutathione and its derivates across the plasma membrane, however the *opt1-3* and *opt6* mutants tested in our study did not exhibit any phenotype difference to WT when exposed to AITC.

Breakdown of glutathione, oxidized glutathione and glutathione S-conjugates happens upon uptake into the vacuole but can also take place in the cytosol and apoplast^56^. As γ-glutamyl transpeptidases (GGTs) and γ-glutamyl peptidases (GGPs) have been implicated in these catabolic processes, we investigated mutants affected in *GGT1* to *GGT4* and *GGP1*, but none showed a higher susceptibility to AITC than WT seedlings when root growth was assessed. The double mutant *pcs1pcs2* impaired in phytochelatin synthesis and the catabolism of glutathione conjugates^61,62^ showed also a WT-like phenotype when exposed to AITC.

Glutathione is an important storage and transport form of reduced sulfur and exerts a negative feedback on sulfate uptake^63^. Glutathione is also involved in the synthesis of GSLs which upon hydrolysis to ITCs release sulfate^3,47^. We tested therefore also mutants impaired in the three genes *SULTR1;2*, *SULTR4;1* and *SULTR4;2* encoding sulfate transporters^64–66^ but did not observe an altered susceptibility to AITC. SULTR1;2 is the major high-affinity sulfate transporter responsible for uptake in Arabidopsis roots while SULTR4;1 and SULTR4;2 mediate the efflux of sulfate from vacuoles to the cytoplasm^64,67^.

## Discussion

In the present study we used a genetic approach to better understand the role of glutathione in the growth inhibition effect of AITC on *A. thaliana* that was indicated by recent studies^17,40,41^.

Glutathione is synthesized in a two-step process through the action of γ-glutamylcysteine synthetase (γ–ECS or GSH1) and glutathione synthetase (GSH2)^42,43^. Several mutants that are impaired in glutathione biosynthesis and accumulate lower levels of glutathione than WT plants under normal growth conditions have been reported. The levels of glutathione depend on the mutation in question, and *nrc1*, *cad2-1*, *pad2-1* and *zir1*, which are affected in GSH1 show glutathione levels that range from 45 to 15% of normal levels^50,51,54,68^. The essential role of glutathione in plant development has been demonstrated by the mutant *rml1* which is impaired in *GSH1* and produces approximately 3% of wild type glutathione levels, leading to extremely short roots^55^. Moreover a null mutant in *GSH1* is embryo lethal^69^. *GSH2* null mutants are lethal at the early seedling stage, but weaker alleles with reduced levels of glutathione, such as *nrc2*^51^, have been described too. All the mutants in GSH1 that we tested, i.e. *pad2-1*, *cad2-1*, *nrc1* and *zir1*, showed reduced root growth under control conditions and a more severe AITC-triggered root inhibition than the WT. Although less pronounced, a similar effect was observed for seedling biomass production. Hence, all these lines impaired in glutathione biosynthesis indicate its important role in AITC-induced physiological responses. The fact that *nrc2*, a mutant affected in GSH2, also exhibited greater sensitivity in its response to AITC confirmed that the root length phenotype was due to the impairment in the glutathione biosynthetic pathway, irrespective of whether *GSH1* or *GSH2* carried the mutation.

It was recently reported that a short-term exposure of *A. thaliana* to AITC (0.01 M) vapours led to a rapid depletion of the cellular glutathione pool and that removing the stressor allowed effective recovery of glutathione levels^40^. Taking this into consideration, we postulate that in our treatment AITC also led to a decrease of glutathione levels. As these were already lower in the mutants compared to the WT this led to stronger phenotypes regarding root length and total biomass production. In addition, as these mutants are affected in glutathione biosynthesis they would respond less efficiently than the WT to an increased demand for glutathione as a consequence of the AITC treatment.

In plants, GSH has been shown to be involved in maintaining the redox homeostasis and mediating signals from the ever-changing environment to intracellular target proteins^70^. That AITC exposure triggered an oxidation and/or depletion of GSH was shown by analysing transgenic lines expressing roGFP2. Both the cytosolic Grx1-roGFP2 and the plastidic TKTP-Grx1-roGFP2 lines exhibited a noticeable decrease in fluorescence intensity when excited at 488 nm following 24 h and 48 h AITC treatments. As we did not observe a clear increase in fluorescence intensity at 405 nm excitation our results indicated that a decrease in glutathione levels was responsible for the lower roGFP2 fluorescence intensity. It has been shown that roGFP2 is almost fully reduced in intact tissues and that a treatment with the GSH1 inhibitor BSO or the electrophilic xenobiotic 1-chloro-2,4-dinitrobenzene led to fully oxidized state of the biosensor accompanied by decreased fluorescence intensity^52^. We cannot exclude the possibility that loss of fluorescence during AITC treatment was due to its interference with the biomarker, but it has been reported that exogenously supplied chemicals responsible for GSH depletion are not likely to interfere with roGFP2^52^.

Our results thus indicated that AITC treatment led to glutathione depletion which contributed to the higher susceptibility of glutathione mutants, but the molecular mechanism behind this physiological response is likely more complicated. We aimed at further depleting the intracellular glutathione levels by applying BSO and AITC simultaneously. The combined treatment resulted in a more severe reduction than the individual treatments for both WT and *pad2-1* in terms of root length and total biomass, indicating an additive effect. However, the two individual treatments had clearly different effects on root growth as e.g. the AITC treatment increased even more the difference for *pad2-1* through the concentration range than for the WT, whereas the reduction between the two highest doses of BSO was less pronounced on the mutant compared to WT. This finding supports the idea that the mutation in *pad2-1* close to the glutamate conjugating residues of GSH1 might influence the affinity of the enzyme for BSO, as it was proposed for *cad2-1*^54,71^. Supplementing the medium with glutathione monoethyl ester in addition to AITC did not diminish the effect of AITC on root growth. This might be due to the oxidation and/or degradation of glutathione over time^72^ or again indicate that the effect triggered by AITC is more complex than a change in glutathione homeostasis.

In order to assess whether the stronger AITC-triggered phenotype of glutathione mutants was specific to this particular GHP or not, mutants were subjected to SFN treatment, which is another isothiocyanate produced by *A. thaliana* accessions^73^. Our results indicated indeed also a higher susceptibility of glutathione mutants than WT towards SFN, which demonstrates that the phenotype induced by AITC is not unique but can be triggered by other ITCs. The assay revealed however also a higher potency for SFN than for AITC, which might be due to differences in physicochemical properties such as their ability to cross the cell membrane, compound stability or differences in reactivity. This is also consistent with our recent work showing that different ITCs lead to various degree of growth inhibition depending on their side chain structure^74^. That SFN injected directly into Arabidopsis leaves has been reported to lead to a rapid decrease of the cellular glutathione pool^75^ further strengthens the involvement of glutathione in ITC-triggered growth inhibition.

In animal cells the fate of ITCs is well characterized: conjugation to glutathione by GSTs resulting in its intracellular depletion and subsequent degradation of dithiocarbamates through enzymatic events catalysed by γ-glutamyltranspeptidase, cysteinylglycinase and *N*-acetyltransferase via the mercapturic acid pathway^76^.

Plants have evolved specific strategies too in order to neutralize potentially harmful compounds such as xenobiotics and heavy metals. As an electrophilic chemical, ITCs might react covalently with the thiol groups of (proteins and) glutathione in plants, similar to what has been demonstrated in different *in vitro* systems^38,77,78^ as well as in insects feeding on GSL or ITC diets^79,80^. This might be one of the possible mechanisms by which AITC leads to lower GSH levels in plants. Conjugation of indolic ITCs with glutathione in the biosynthesis of indole-sulfur phytoalexins and in PEN2-mediated indole GSL hydrolysis has been reported recently^35,81^. Conjugation of aliphatic ITCs with glutathione, the further processing of these conjugates and the molecular mechanism of ITC-triggered reduction of glutathione levels *in planta* still requires further investigation. A conjugation of AITC to glutathione *in planta* might be non-enzymatic or catalysed by GSTs, as indicated by the AITC-induced upregulation of several GST-encoding genes^17,40,41^ and similar to what was recently shown for indol-3-ylmethyl-ITC^35^. Glutathione S-conjugates are usually sequestered by distinct ABC transporters from the cytosol into the vacuole for degradation, although breakdown of glutathione, oxidized glutathione and glutathione S-conjugates also happens outside the vacuole^56^. We aimed at investigating the possible intracellular fate of glutathione-ITC conjugates by analysing different mutants that might be involved in the processing and/or transport of these compounds. However, the chosen mutants impaired in ABC-type MRP transporters, oligopeptide transporters (OPTs) and γ-glutamyl transpeptidases (GGTs) did not exhibit a difference in root growth inhibition during AITC treatment compared to the WT. This suggests that these proteins do not play a major role in the transport and breakdown of putative glutathione-AITC conjugates. Alternatively functional redundancy might have prevented us from observing any phenotype for single mutants. Hence, further studies are needed to elucidate the fate of AITC in plant tissue.

GSLs, the precursors of ITCs, constitute in addition to their role in defence responses a storage for reduced sulfur as well as a good example on how the partitioning of this macronutrient is controlled between primary and secondary metabolism^82^. Glutathione is also an important storage and transport form of reduced sulfur owing to the thiol-containing Cys residue incorporated into the tripeptide and its biosynthesis is strongly dependent on the sulfur assimilatory pathway including necessary feedback inhibition mechanisms^63^. A mutant impaired in the major high-affinity sulfate transporter SULTR1;2 responsible for uptake in Arabidopsis roots was reported earlier to contain decreased amounts of glutathione^83^ but our data indicate that *sultr1;2* seedlings exposed to AITC have a WT-like response. The *sultr4;1* and *sultr4;2* mutants impaired in mediating the efflux of sulfate from vacuoles to the cytoplasm^67^ displayed also WT-like growth under AITC treatment. Taking into consideration that mutants affected in glutathione biosynthesis exhibited a susceptible phenotype while, under our conditions, none of the tested sulfate transporter mutants did so, the latter did not seem to affect glutathione homeostasis sufficiently to result in increased susceptibility to AITC.

In the present study we provided genetic evidence about the important role of GSH in AITC-induced growth inhibition. Our analysis of a series of mutants has revealed that those directly impaired in glutathione biosynthesis exhibited a higher degree of growth reduction than WT. Our results indicate glutathione depletion upon treatment with AITC, corroborating earlier studies^17,40,75^. The underlying molecular mechanism of AITC-induced physiological responses -especially the consequences of GSH depletion- seems to be complex. Glutathione plays an essential role in the control of root development via cell cycle regulation as it was comprehensively demonstrated in the *rml1* mutant^55^. Interestingly, previous transcriptional analysis uncovered that AITC treatment affected the expression profile of certain genes involved in the regulation of cell cycle^23^, indicating the phytochemical-induced growth inhibition might be an indirect consequence of cell cycle misregulation due to GSH depletion. According to our current knowledge the most likely scenario is that AITC treatment causes ROS production^19,20^ and induces the expression of several GSTs^17,40,41^ as part of the plant’s detoxification system to conjugate AITC to glutathione leading to its intracellular depletion. Recruitment of glutathione from the cytosol into the nucleus is essential at the beginning of cell proliferation to provide proper redox environment^84,85^. However, the import of glutathione into the nucleus under normal physiological conditions occurs at the expense of the cytosolic pool^85^ which together with AITC generates an extreme demand for glutathione. Plants have to adjust their metabolism in order to supply enough glutathione for AITC neutralization and in the same time to provide enough redox buffer for the nucleus to maintain proper cell proliferation. However, based on our results mutants abolished in glutathione biosynthesis failed to accomplish this mission. The appropriate glutathione supply within different subcellular compartments is necessary to maintain the physiological redox balance within the cell^86^. Although in our study roGFP2 expressed in the cytosol or in the plastid exhibited reduced fluorescence intensity upon AITC treatment, it is currently not known if AITC can hijack glutathione only in the cytosol and therefore interrupt the nuclear import, or is able to deplete different pools at the same time. Is AITC able to deplete directly the nuclear glutathione pool? The nuclear glutathione pool is more resistant to depletion by BSO than the cytosolic as it was demonstrated in 3T3 fibroblasts and other human cell lines^87,88^, hence we cannot exclude the possibility that AITC -which presumably acts differently from BSO- is able to deplete efficiently the nuclear pool. Depletion of glutathione affects root development also by interfering with auxin transport^84,89,90^. Although transcriptional analysis of AITC-treated seedlings revealed down-regulation of many auxin-inducible genes, auxin marker lines exposed to AITC did not indicate a change in auxin response and auxin-resistant mutants did not reveal an altered resistance to AITC^74^. Whether alternative pathways such as thioredoxins^90^ or other members of the redox hub such as ascorbate^44^ might be involved in the process also remains to be elucidated. Moreover, the possibility that AITC *in planta* can interact with oxidized glutathione -as it was shown *in vitro*^77^- in addition to the reduced form also needs to be investigated. The identification of additional members involved in the AITC-induced responses is a key step to fully understand the complexity of this physiological process.

## Methods

### Chemicals

Allyl-isothiocyanate (AITC, CAS 57-06-7; 377430; 95%), sulforaphane (SFN, CAS 142825-10-3; S6317; 95%), dimethyl sulfoxide (DMSO, CAS 67-68-5; D8418) and L-buthionine-sulfoximine (BSO, CAS 83730-53-4; B2515) were purchased from Sigma-Aldrich. Propidium iodide (PI, CAS 25535-16-4; Molecular Probes P3566) was purchased from Thermo Fisher Scientific.

### Plant material and growth conditions

Seeds of *A. thaliana* mutant lines used in our study were ordered from the European Arabidopsis Stock Centre (NASC) or kindly donated by authors (see Supplementary Table S1 for further details). Seeds were surface sterilised using chlorine gas for 4 h, and then stratified for at least 3 days at 4 °C in water. Seeds were sown on square plates (Greiner Bio One; 120 × 120 × 17 mm) containing 75 ml solid ½ strength Murashige-Skoog medium (pH 5.7) (Sigma-Aldrich, M5524 supplemented with 2% sucrose and 1% agar). Plates were sealed and placed in a vertical position into a controlled growth chamber (VB1514 Vötsch Industrietechnik) under a 16 h photoperiod (75 µmol m^−2^ s^−1^) at 22 °C/18 °C for 10 days.

### Validation of mutant lines

All mutants were either homozygous for the mutation when obtained or propagated to homozygosity. The homozygosity of T-DNA insertion mutants was verified by PCR using T-DNA specific and target specific primers (Supplementary Table S2). In case of ethyl methanesulfonate mutants, the region surrounding the expected point mutation was amplified by PCR and the single point mutation was verified by sequencing. Genotyping results of the mutants are presented in Supplementary Figure S5.

### Treatment with ITCs and BSO

*A. thaliana* seedlings were exposed to ITC for 10 days while growing vertically on square plates containing solid *in vitro* medium as described above. Seeds were sown in parallel on medium supplemented with ITC, which was added just before pouring the plates, and on control plates (medium without ITC). The AITC concentrations used were 50 µM, 100 µM, 300 µM and 500 µM, based on our recent study on the effect of AITC on root growth inhibition^74^. Selected mutants were also exposed to SFN (30 mM stock solution prepared in DMSO) at the concentrations of 10 µM, 25 µM, 50 µM and 100 µM. In this case control plates consisted of DMSO-supplemented medium. Unless stated otherwise four replicate plates were prepared for each treatment and mutant seedlings were grown together with WT seedlings on each plate.

BSO treatment was performed similarly as for ITCs by adding it (100 mM stock solution prepared in water) to the medium (either on its own or in combination with ITC) to obtain BSO concentrations ranging from 50 µM to 700 µM.

### Measurement of growth parameters

Primary root length and total seedling biomass were measured in order to evaluate the ITC-triggered growth inhibition of mutants compared to WT. Pictures of vertically grown seedlings were taken at three different time points (4, 7 and 10 days) after sowing out, and primary root length was measured using the ImageJ software^91^. Total biomass of the seedlings was measured at day 10. Seeds that did not germinate or develop were not taken into consideration when calculating the average root length or biomass. Statistical significance testing was done by Student’s t-test using SigmaPlot (Systat Software; ver. 13.0).

### Confocal laser scanning microscopy (CLSM) analysis

Seeds of two transgenic lines expressing redox-sensitive GFP2 (roGFP2) either in the cytosol (Grx1-roGFP2) or the plastids (TKTP-Grx1-roGFP2)^92^ and Col-0 WT seedlings were sown onto 6-well cell culture plates (Sigma-Aldrich, CLS3516) containing 3 ml of liquid ½ MS medium (pH 5.7) supplemented with 2% sucrose. Plates were sealed and placed into a controlled growth chamber for 7 days under the same conditions as mentioned above. On day 7 seedlings were transferred to fresh growth medium, and then some of the plates were supplemented with AITC to a final concentration of 1 mM whereas others remained non-treated (control). Plates were sealed and placed back into the growth cabinet.

Samples from two replicate wells were investigated after 24 h, 48 h and 72 h of AITC treatment. Prior to imaging, seedlings were rinsed in sterile water and stained with 500 nM propidium iodide (PI, 50 µM stock solution in H_2_O) for 1 minute, then rinsed again with water to get rid of the excess staining. PI was used as a marker to monitor the cell viability during live cell imaging. Samples were mounted onto glass slides using water as a medium and investigated under a Leica (DMI 6000 CS AFC Bino inverted microscope) TCS SP8 instrument. The following settings were applied in our analysis: HCX IRAPO L 25x/0.95 NA (FWD = 2.4 mm) water immersion objective; 1 airy unit pinhole size; sequential scanning between frames; UV (405 nm) and white light (WLL 470–670 nm) lasers. GFP was excited at 405/488 nm and detected at 498–530 nm, whereas PI was excited at 561 nm and detected between 571 and 715 nm. Leica HyD detectors were used in standard mode for recording the signal of fluorophores. Images were processed by Leica Application Suite X (LAS X 2.0), Adobe Photoshop CC and Adobe Illustrator CC softwares.

## Electronic supplementary material

Supplementary Figures and Tables
