# Supplementary material for: Arabidopsis mutants impaired in glutathione biosynthesis exhibit higher sensitivity towards the glucosinolate hydrolysis product allyl-isothiocyanate
Source: Sci Rep. 2018 Jun 28;8:9809. doi: 10.1038/s41598-018-28099-1 (PMC6023892; doi:10.1038/s41598-018-28099-1)
Supplement: Supplementary file 1 — Supplementary Figures and Tables [file 41598_2018_28099_MOESM1_ESM.pdf]

Arabidopsis mutants impaired in glutathione biosynthesis exhibit higher sensitivity towards the glucosinolate hydrolysis product allyl-isothiocyanate

János Urbancsok, Atle M Bones & Ralph Kissen*

Cell, Molecular Biology and Genomics Group, Department of Biology, Norwegian University of Science and Technology, NO-7491 Trondheim, Norway

* corresponding author

Ralph Kissen

E-mail: ralph.kissen@bio.ntnu.no

Tel.: (+47) 73596229

Supplementary Figure S1. The ratio between the mean root length under a given AITC concentration and that of the control treatment calculated for 10 day old seedlings of the Col-0 WT and the glutathione mutants (A) *cad2-1*, (B) *zir1*, (C) *nrc1*, (D) *nrc2*.

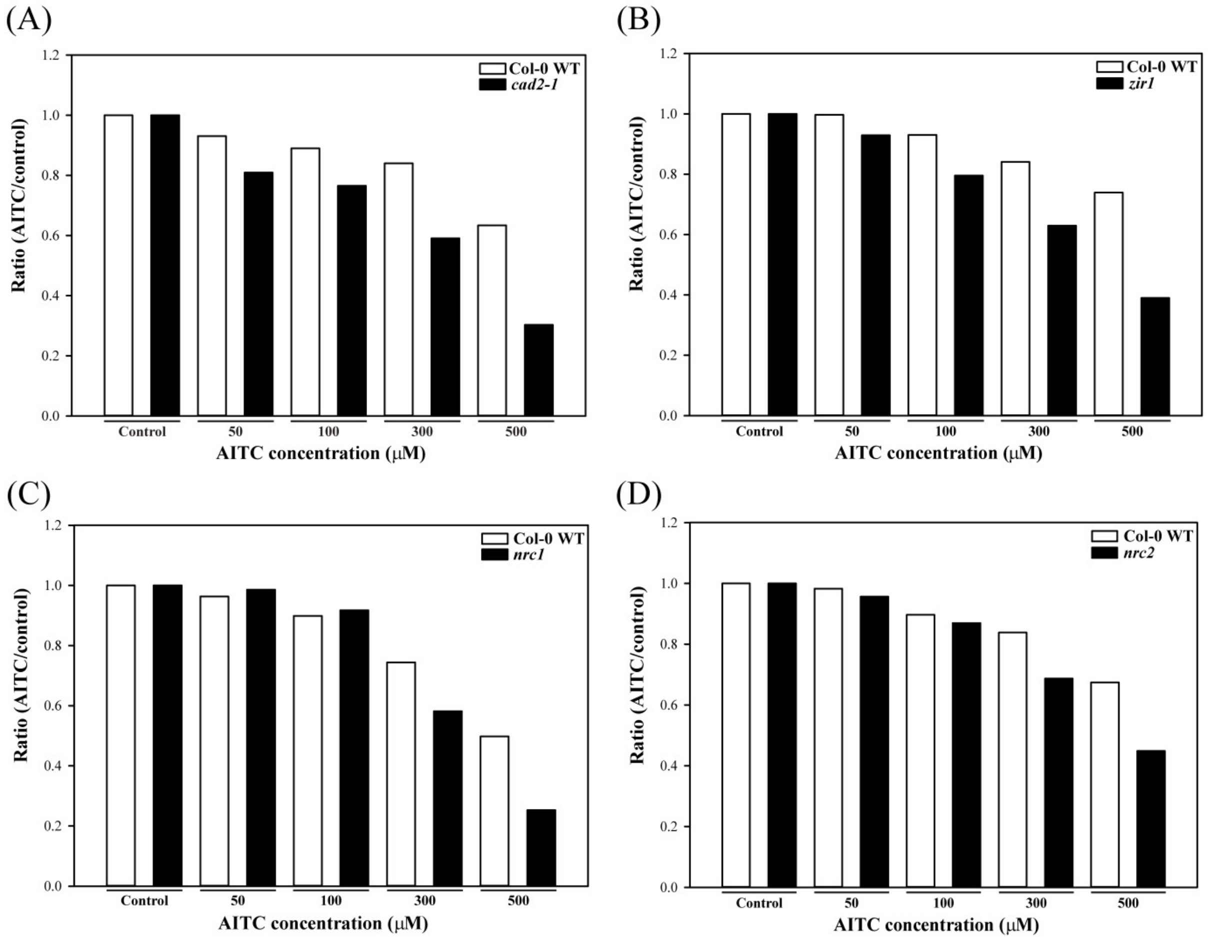

Supplementary Figure S2. Root elongation analysis over time using WT and glutathione mutants grown either on control medium or on medium supplemented with 300 µM AITC: (A) *pad2-1*, (B) *cad2-1*, (C) *zir1*, (D) *nrc1* and (E) *nrc2*. Root lengths were measured (n= 48 for mutants; n= 96 for WT) at each time point (at day 4, 7 and 10) and the mean values were used to calculate the average root length increment. Note that the values for WT are identical for each of the panels as all seedlings were grown in parallel.

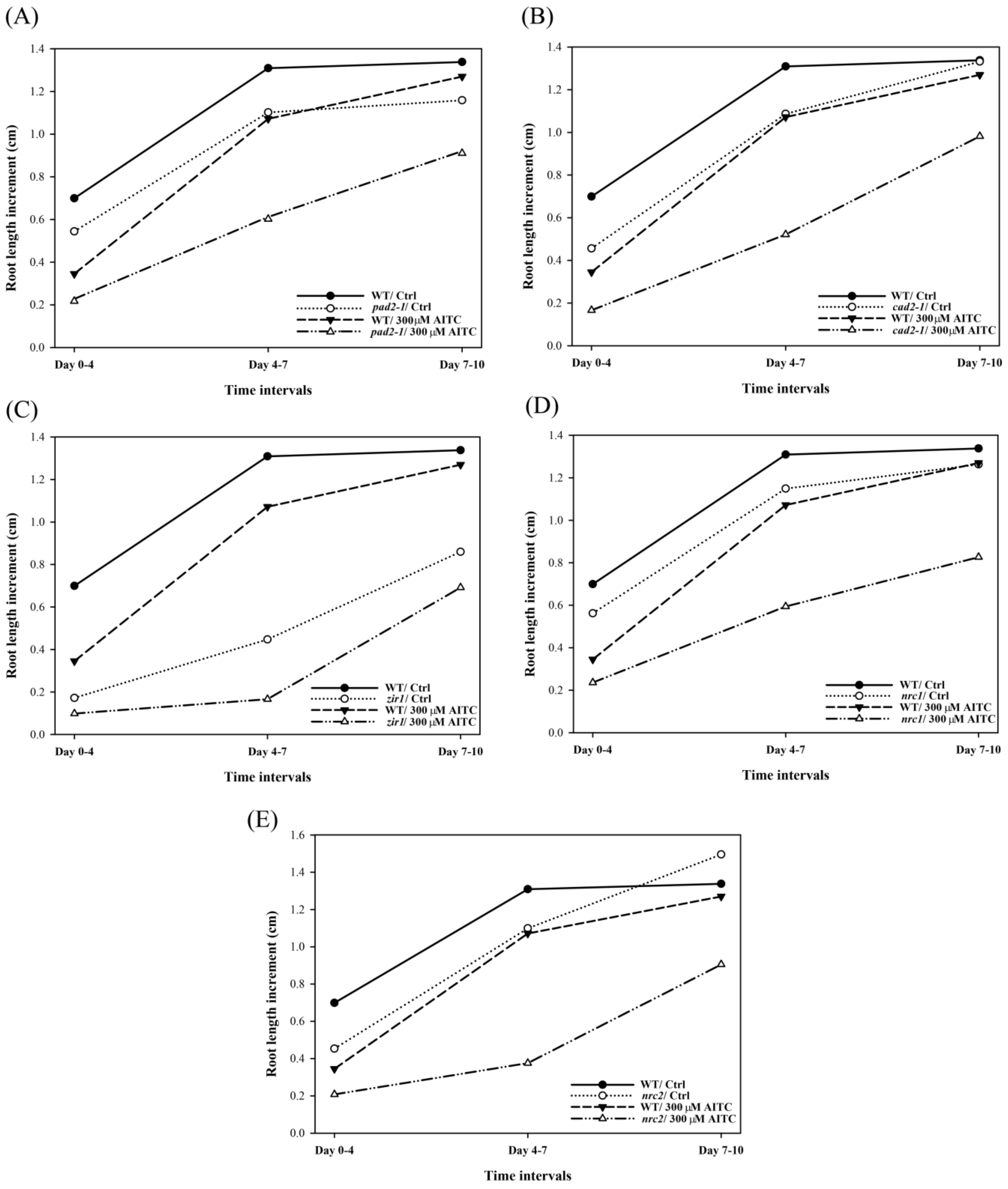

Supplementary Figure S3. The ratio between the mean biomass under a given AITC concentration and that of the control treatment calculated for 10 day old seedlings of the Col-0 WT and the glutathione mutants (A) *cad2-1*, (B) *zir1*, (C) *nrc1*, (D) *nrc2*.

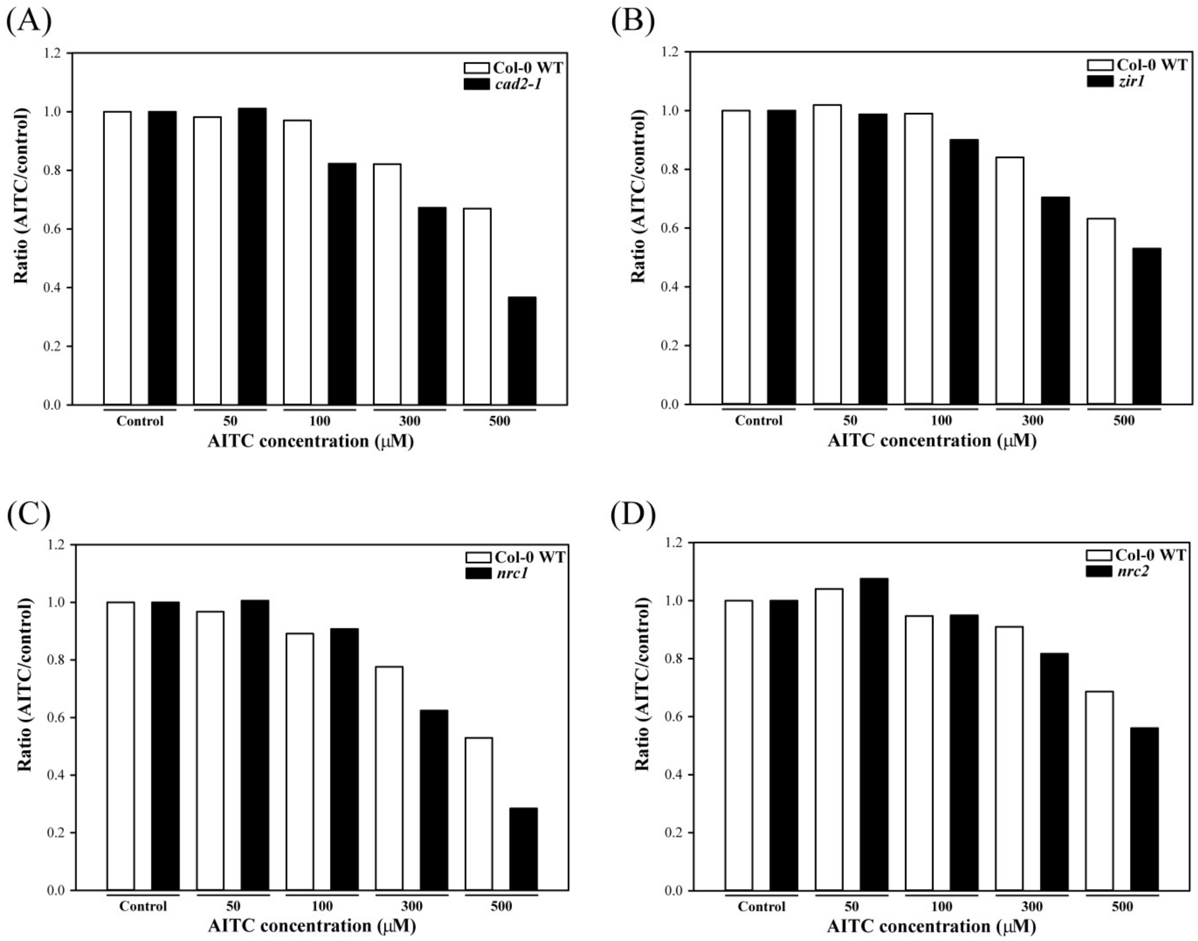

Supplementary Figure S4. Ratios of (A) mean root length and (B) mean biomass between a given treatment (AITC, BSO or AITC+BSO) and that of the control treatment calculated for 10 day old seedlings of the Col-0 WT and the glutathione mutant p*ad2-1*.

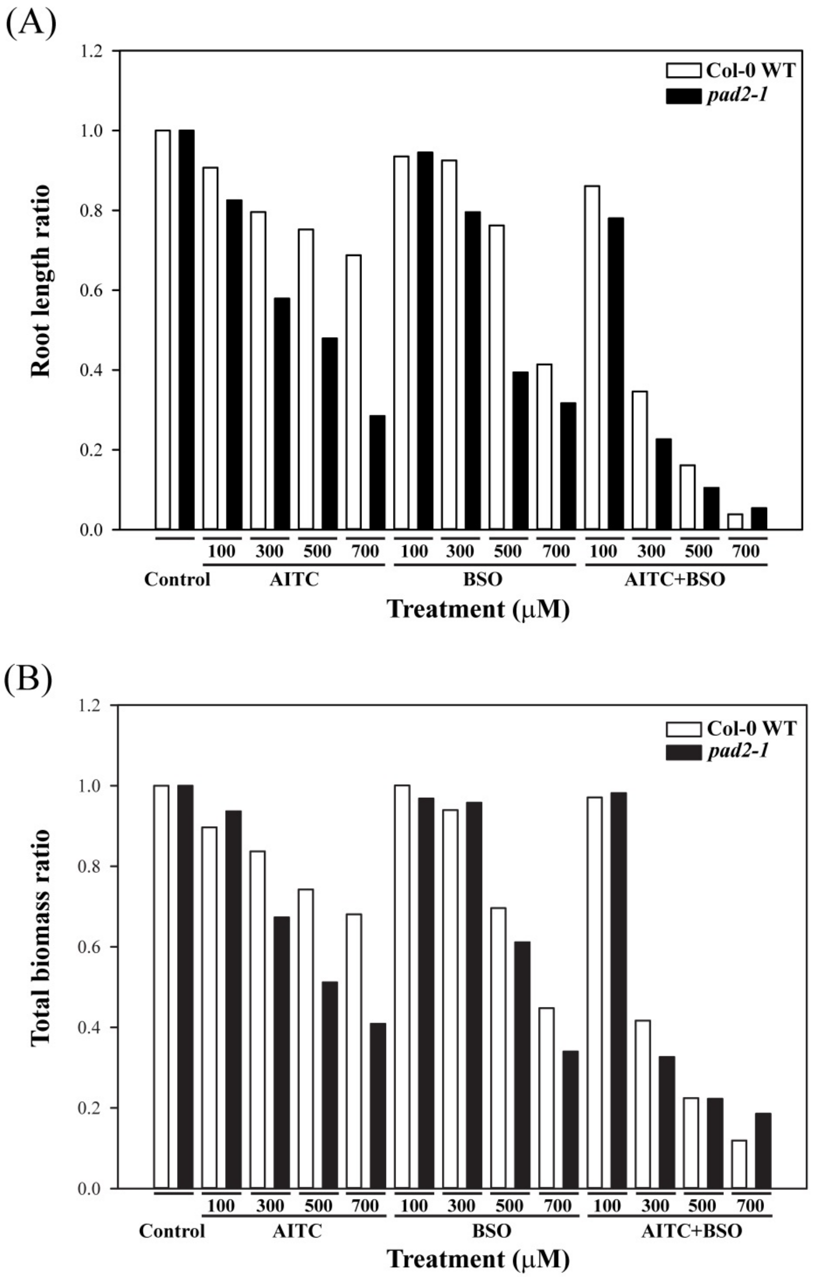

Supplementary Figure S5. Genotyping results of mutants used in this study (see also Supplementray Table S1) performed as described in more detail in the Methods section using the primers listed in Supplementary Table S2.

(A) *GSH1* deficient mutants *pad2-1*, *cad2-1*, *zir1* and *nrc1*

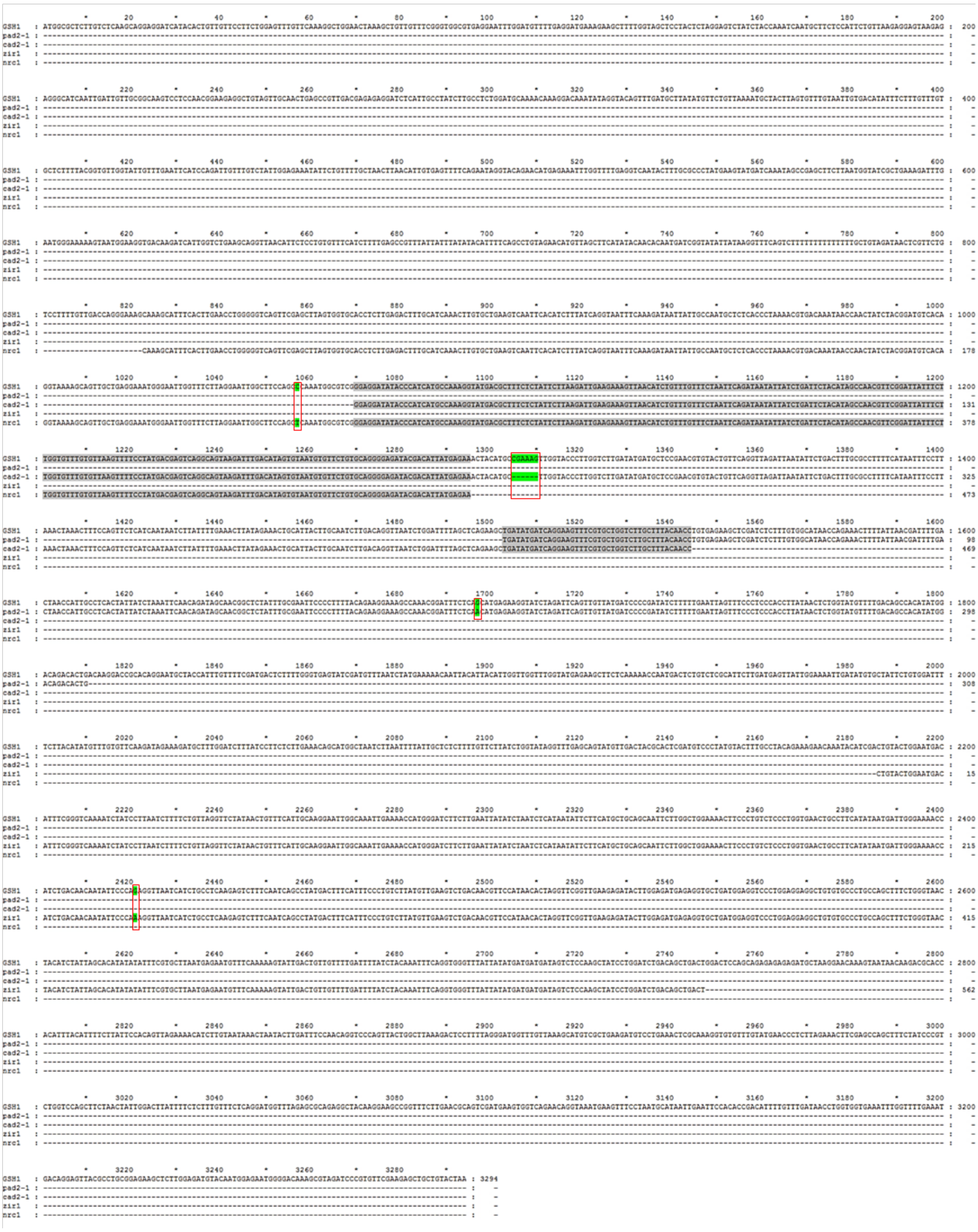

(B) *GSH2* deficient mutant *nrc2*

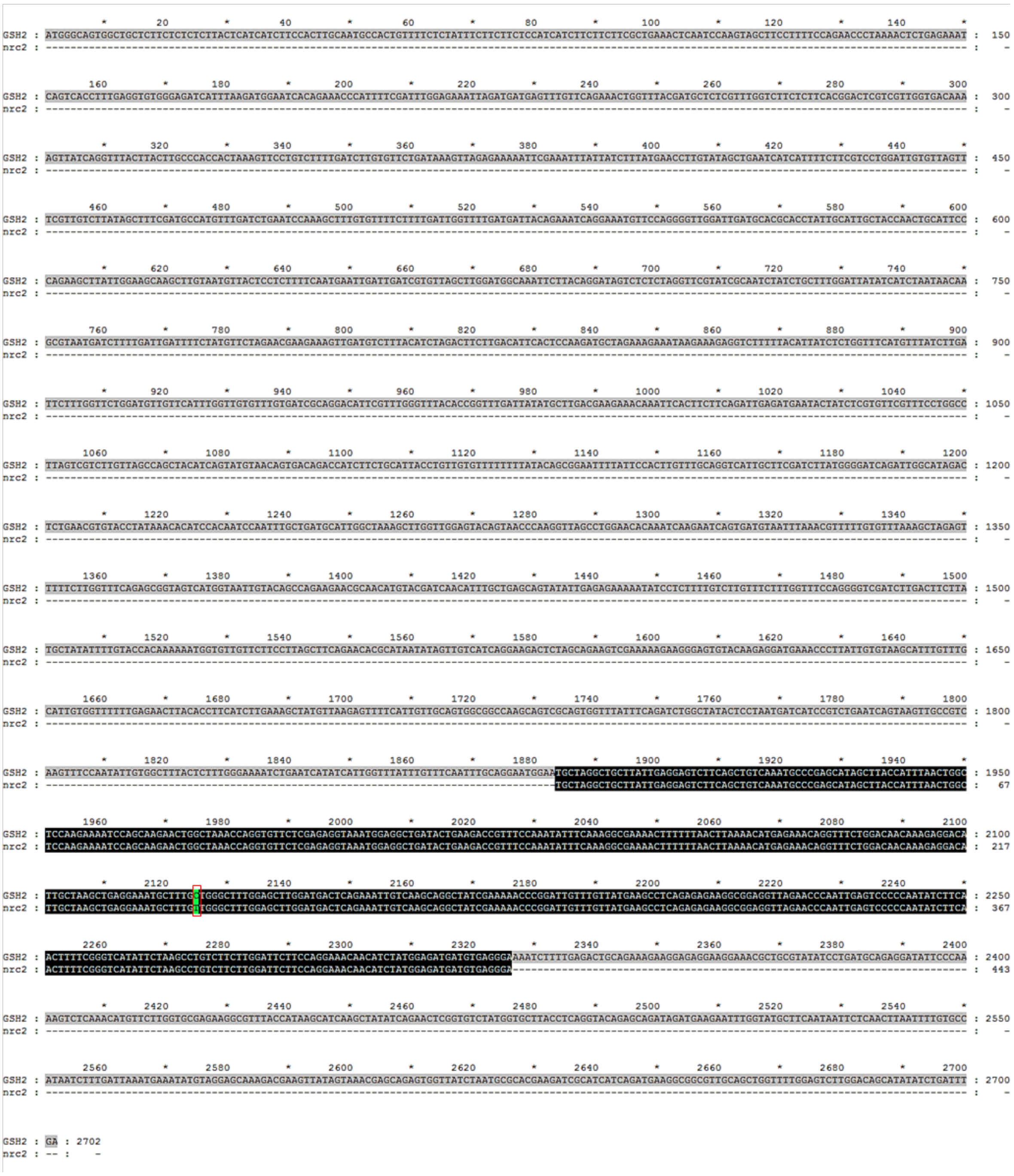

(C) ***ggp1-1*** (GK-319F10)

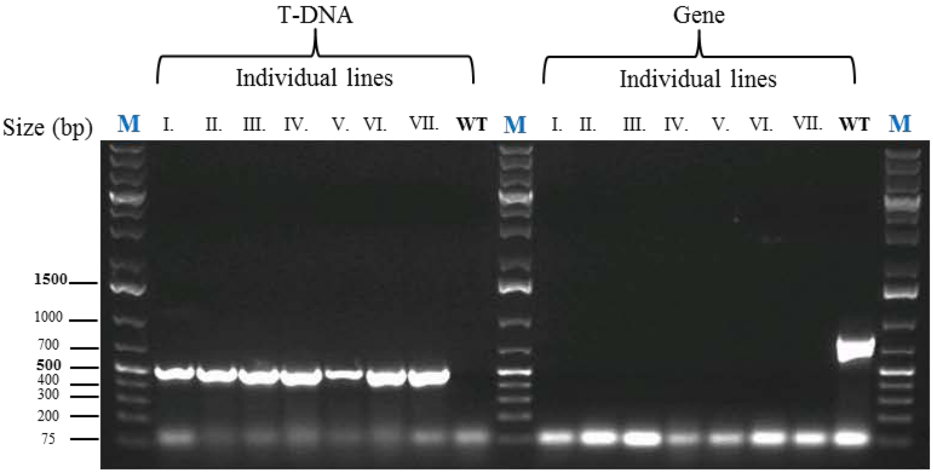

(D) ***ggt1-1*** (SAIL_1178_C01)

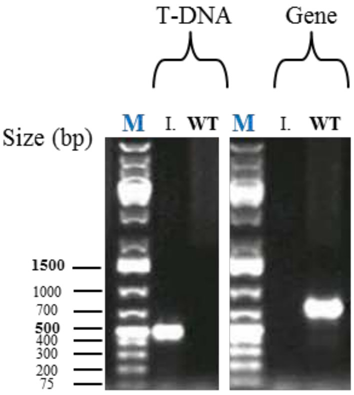

(E) ***ggt2-1*** (SAIL_6_G02)

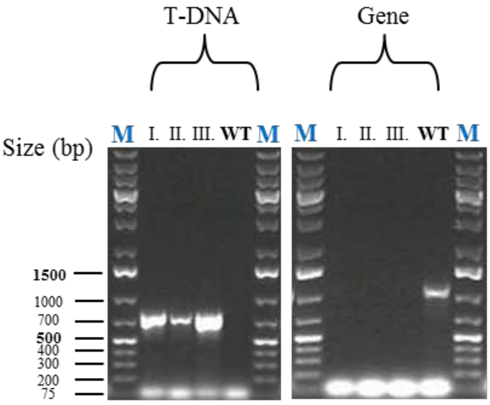

(F) ***ggt3*** (ET1113)

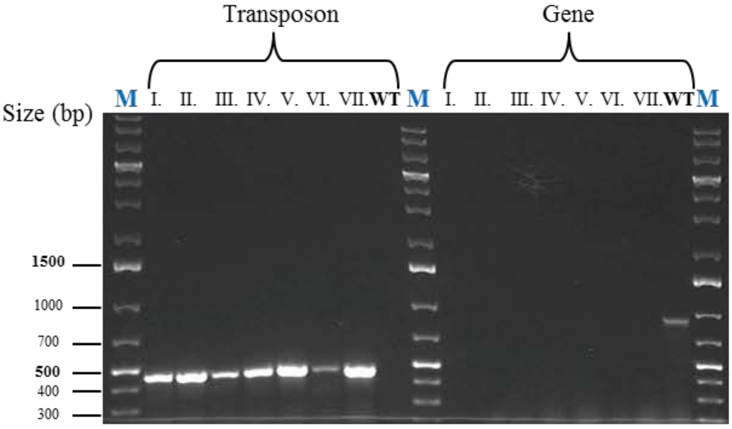

(G) ***ggt4*** (GT_5_57895)

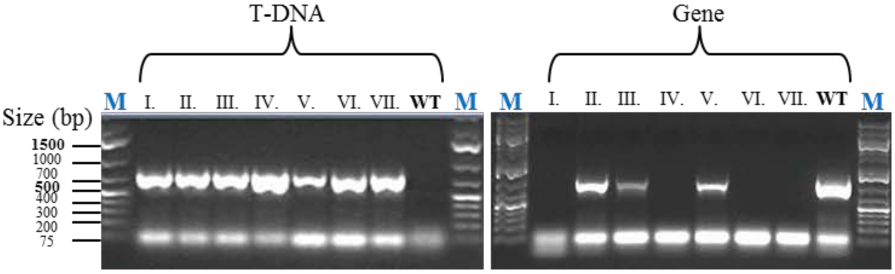

(H) ***mrp1*** (SALK_017431)

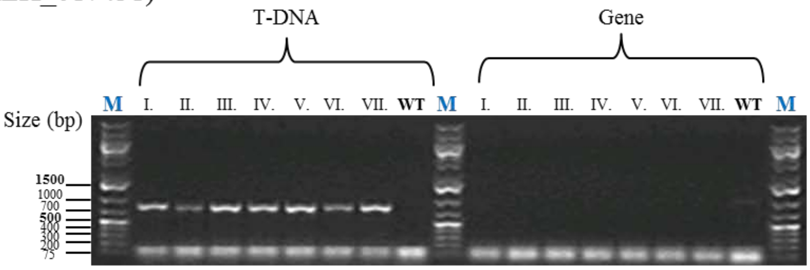

(I) ***mrp2-2*** (SALK_127425)

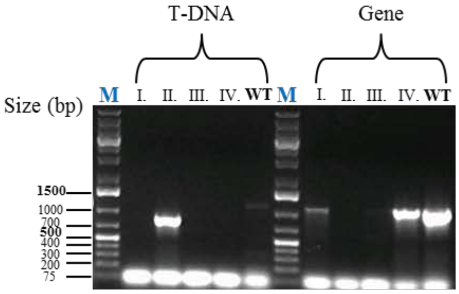

(J) ***mrp3*** (SALK_044022)

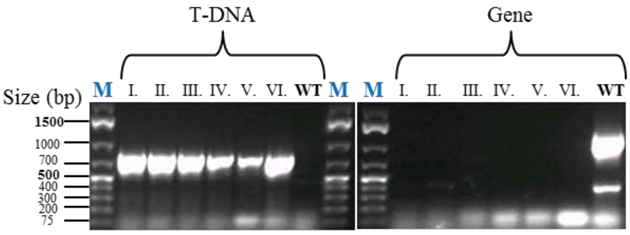

(K) ***opt1-3*** (SALK_052985)

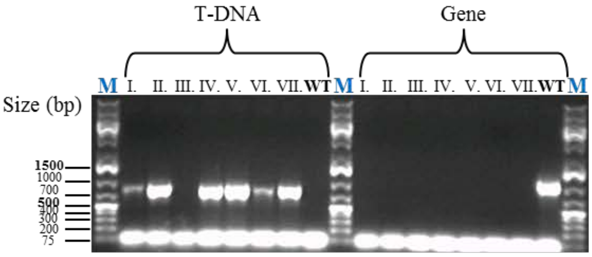

(L) ***opt6*** (SALK_201534)

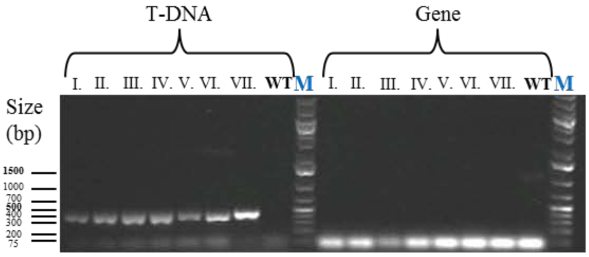

(M) ***sultr1;2*** (SM_3.30250)

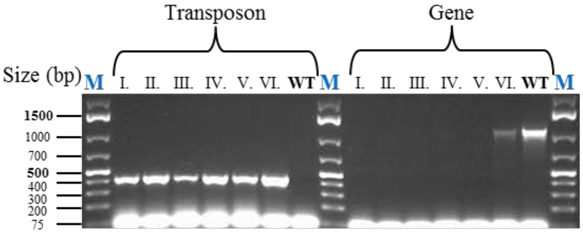

(N) ***sultr4;1*** (SALK_103873)

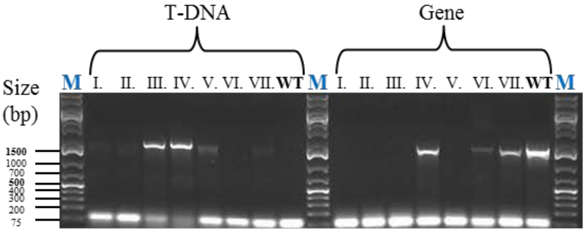

(O) ***sultr4;2*** (SALK_103827)

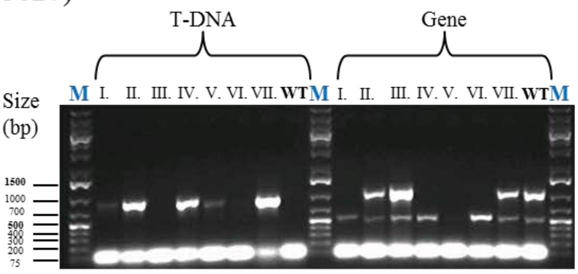

Supplementary Table S1. List of mutants used in our study. Mutants that are underlined in the table showed an increased susceptibility to the AITC treatment.

Mutant name	Mutant ID	Source/ Stock ID	Gene name	Gene ID	Description	Reference
<u>cad2-1</u>		Cobbett	*GSH1/CAD2*	AT4G23100	*cadmium sensitive 2* ; γ-ECS/GSH1 is the first and rate-limiting enzyme of glutathione biosynthesis	1
clt1clt2clt3		Cobbett	*CLT1*<br>*CLT2*<br>*CLT3*	AT5G19380<br>AT4G24460<br>AT5G12170	*chloroquine-resistance transporter-like transporters 1, 2, 3* ; transporters involved in glutathione transport from plastid to cytosol	2
ggp1-1	GK-319F10	N430598	*GGP1*	AT4G30530	*γ-glutamyl peptidase 1* ; GGP1 hydrolyses γ-glutamyl peptide bonds	3
ggt1-1	SAIL_1178_C01	Ren	*GGT1*	AT4G39640	*γ-glutamyl transpeptidase 1* ; GGT1 plays a role in the degradation of glutathione and its S-conjugates	4
ggt2-1	SAIL-6-G02	N862221	*GGT2*	AT4G39650	*γ-glutamyl transpeptidase 2* ; GGT2 is involved in the degradation of glutathione and its S-conjugates	5
ggt3	ET1113	N26010	*GGT3*	AT1G69820	*γ-glutamyl transpeptidase 3* ; GGT3 plays role in the degradation of glutathione and its S-conjugates	6
ggt4	GT_5_57895	Grill	*GGT4*	AT4G29210	*γ-glutamyl transpeptidase 4* ; GGT4 is involved in the degradation of glutathione and its S-conjugates	7
mrp1	SALK_017431	N685353	*MRP1/ABCC1*	AT1G30400	*multidrug resistance-associated protein 1* ; glutathione S-conjugate pump	8
mrp2-2	SALK_127425	N627425	*MRP2/ABCC2*	AT2G34660	*multidrug resistance-associated protein 2* ; glutathione S-conjugate pump	9
mrp3	SALK_044022	N662277	*MRP3/ABCC3*	AT3G13080	*multidrug resistance-associated protein 3* ; glutathione S-conjugate and chlorophyll catabolite transporter	10
<u>nrc1</u>		Schroeder	*GSH1*	AT4G23100	*non response to cadmium 1* ; γ-ECS/GSH1 is the first and rate-limiting enzyme of glutathione biosynthesis	11
<u>nrc2</u>		Schroeder	*GSH2*	AT5G27380	*non response to cadmium 2* ; glutathione synthetase is the second enzyme of glutathione biosynthesis	11

opt1-3	SALK_052985	N662479	*OPT1*	AT5G55930	*oligopeptide transporter 1*	12
opt6	SALK_201534	N689065	*OPT6*	AT4G27730	*oligopeptide transporter 6* ; involved in the transport of glutathione derivatives	13
pad2-1		N3804	*GSH1*	AT4G23100	*phytoalexin deficient 2* ; γ-ECS/GSH1 is the first and rate-limiting enzyme of glutathione biosynthesis	14
pcs1pcs2		Grill	*PCS1*<br>*PCS2*	AT5G44070<br>AT1G03980	*phytochelatin synthase 1 and 2* ; synthesis of phytochelatins and catabolism of glutathione conjugates	15
sultr1;2	SM_3.30250	N116961	*SULTR1;2/SEL 1*	AT1G78000	*sulfate transporter 1;2* ; high-affinity sulfate transporter	16
sultr4;1	SALK_103873	N673083	*SULTR4;1*	AT5G13550	*sulfate transporter 4;1* ; low-affinity sulfate transporter	17
sultr4;2	SALK_103827	N676417	*SULTR4;2*	AT3G12520	*sulfate transporter 4;2*; low-affinity sulfate transporter	18
zir1		Yeh	*GSH1*	AT4G23100	*zinc tolerance induced by iron 1* ; ECS/GSH1 is the first and rate-limiting enzyme of glutathione biosynthesis	19

References

1 Cobbett, C. S., May, M. J., Howden, R. & Rolls, B. The glutathione-deficient, cadmium-sensitive mutant, *cad2–1*, of *Arabidopsis thaliana* is deficient in γ-glutamylcysteine synthetase. *Plant J.* **16**, 73-78, (1998).

2 Maughan, S. C. *et al.* Plant homologs of the *Plasmodium falciparum* chloroquine-resistance transporter, PfCRT, are required for glutathione homeostasis and stress responses. *Proc. Natl. Acad. Sci. U.S.A.* **107**, 2331-2336, (2010).

3 Geu-Flores, F. *et al.* Cytosolic γ-glutamyl peptidases process glutathione conjugates in the biosynthesis of glucosinolates and camalexin in Arabidopsis. *Plant Cell* **23**, 2456-2469, (2011).

4 Su, T. *et al.* Glutathione-indole-3-acetonitrile is required for camalexin biosynthesis in *Arabidopsis thaliana*. *Plant Cell* **23**, 364-380, (2011).

5 Ohkama-Ohtsu, N. *et al.* Characterization of the extracellular γ-glutamyl transpeptidases, GGT1 and GGT2, in Arabidopsis. *Plant J.* **49**, 865-877, (2007).

6 Martin, M. N., Saladores, P. H., Lambert, E., Hudson, A. O. & Leustek, T. Localization of members of the γ-glutamyl transpeptidase family identifies sites of glutathione and glutathione S-conjugate hydrolysis. *Plant Physiol.* **144**, 1715-1732, (2007).

7 Blum, R., Meyer, K. C., Wunschmann, J., Lendzian, K. J. & Grill, E. Cytosolic action of phytochelatin synthase. *Plant Physiol.* **153**, 159-169, (2010).

8 Song, W. Y. *et al.* Arsenic tolerance in Arabidopsis is mediated by two ABCC-type phytochelatin transporters. *Proc. Natl. Acad. Sci. U.S.A.* **107**, 21187-21192, (2010).

9 Frelet-Barrand, A. *et al.* Comparative Mutant Analysis of Arabidopsis ABCC-Type ABC Transporters: AtMRP2 Contributes to Detoxification, Vacuolar Organic Anion Transport and Chlorophyll Degradation. *Plant Cell Physiol.* **49**, 557-569, (2008).

10 Tommasini, R. *et al.* An ABC-transporter of *Arabidopsis thaliana* has both glutathione-conjugate and chlorophyll catabolite transport activity. *Plant J.* **13**, 773-780, (1998).

11 Jobe, T. O. *et al.* Feedback inhibition by thiols outranks glutathione depletion: a luciferase-based screen reveals glutathione-deficient γ-ECS and glutathione synthetase mutants impaired in cadmium-induced sulfate assimilation. *Plant J.* **70**, 783-795, (2012).

12 Hofstetter, S. S., Dudnik, A., Widmer, H. & Dudler, R. Arabidopsis YELLOW STRIPE-LIKE7 (YSL7) and YSL8 transporters mediate uptake of Pseudomonas virulence factor syringolin A into plant cells. *Mol. Plant-Microbe Interact.* **26**, 1302-1311, (2013).

13 Cagnac, O., Bourbouloux, A., Chakrabarty, D., Zhang, M.-Y. & Delrot, S. AtOPT6 transports glutathione derivatives and is induced by primisulfuron. *Plant Physiol.* **135**, 1378-1387, (2004).

14 Glazebrook, J. & Ausubel, F. M. Isolation of phytoalexin-deficient mutants of Arabidopsis thaliana and characterization of their interactions with bacterial pathogens. *Proc. Natl. Acad. Sci. U.S.A.* **91**, 8955-8959, (1994).

15 Blum, R. *et al.* Function of phytochelatin synthase in catabolism of glutathione-conjugates. *Plant J.* **49**, 740-749, (2007).

16 Shibagaki, N. *et al.* Selenate-resistant mutants of *Arabidopsis thaliana* identify Sultr1;2, a sulfate transporter required for efficient transport of sulfate into roots. *Plant J.* **29**, 475-486, (2002).

17 Zuber, H. *et al.* Sultr4;1 mutant seeds of Arabidopsis have an enhanced sulphate content and modified proteome suggesting metabolic adaptations to altered sulphate compartmentalization. *BMC Plant Biol.* **10**, 1-13, (2010).

18 El Kassis, E. *et al.* Characterization of a selenate-resistant Arabidopsis mutant. Root growth as a potential target for selenate toxicity. *Plant Physiol.* **143**, 1231-1241, (2007).

19 Shanmugam, V., Tsednee, M. & Yeh, K.-C. ZINC TOLERANCE INDUCED BY IRON 1 reveals the importance of glutathione in the cross-homeostasis between zinc and iron in *Arabidopsis thaliana*. *Plant J.* **69**, 1006-1017, (2012).

Supplementary Table S2. List of primers used for validation of the mutant lines tested in our study. (For: forward gene specific primer, Rev: reverse gene specific primer; T-DNA: primer annealing on T-DNA)

Mutant name	Primer sequence (5′-3′)
cad2-1	For GGAGGATATACCCATCATGC
	Rev GGTTGTAAAGCAAGACCAGC
ggp1-1	For AGAATGATGATTGGATCCTTAAGC
	Rev GATCGCTATGCTATCAGGAATCTC
	T-DNA ATAATAACGCTGCGGACATCTACATTTT
ggt1-1	For GACTTGGCCTCAACGTATACATC
	Rev CGCGAGACTTAACAATACTTTGC
	T-DNA TAGCATCTGAATTTCATAACCAATCTCGATACAC
ggt2-1	For CTCCCACCATTGTACTCAAGG
	Rev CTAGAGGCGGAGTATCCAAGG
	T-DNA TAGCATCTGAATTTCATAACCAATCTCGATACAC
ggt3	For GTATCACTTCTTGGTCACACTGC
	Rev GATCAAATAACTTACCTGCCACC
	T-DNA CCGTTTTGTATATCCCGTTTCCGT
ggt4	For AATCGGTGGTGGTTCTTTCTTGATT
	Rev TGTTGAGAAATCATCCATCTCGTTG
	T-DNA ACCCGACCGGATCGTATCGGT
mrp1	For ATTGGGGGTTTTATGTGAAGC
	Rev ACTTCACTGTATCCATTGCCG
	T-DNA TGGTTCACGTAGTGGGCCATCG
mrp2-2	For GCTTTTCTGCGTTTGTAGTGG
	Rev TTTTCCCAAGCATAACACCTG
	T-DNA TGGTTCACGTAGTGGGCCATCG
mrp3	For AAGTCAGCAATTGCTTTGGAG
	Rev AGGGAGCCTTTTTATGCTCAC
	T-DNA TGGTTCACGTAGTGGGCCATCG
nrc1	For CAAAGCATTTCACTTGAACC
	Rev TTCTCATAATGTCGTATCTCCC
nrc2	For TGCTAGGCTGCTTATTGAGG
	Rev TCCCTCACATCATCTCCATAG
opt1-3	For AAAACCAAAGAAAACGGATCC
	Rev GACTATTACCCACAACAACAAAATG
	T-DNA TGGTTCACGTAGTGGGCCATCG
opt6	For AAAATTCAAACATTACAAGCCATG
	Rev GATAGGGAAGGTCTTGGCATC
	T-DNA TGGTTCACGTAGTGGGCCATCG
pad2-1	For TGATATGATCAGGAAGTTTCG
	Rev CAGTGTCTGTCCATATGTGG
pcs1pcs2	For_{pcs1} CGAATCCACTAACGAATCTTCC
	Rev_{pcs1} TTGATTCATCAAACCACCTCC
	For_{pcs2} GGTTCACGAATTATTGATAGCC
	Rev_{pcs2} CTCTGTTCTTCGAATCTCAGC
	T-DNA ATGGCGAAATCAAGGCATC
sultr1;2	For ATTGGTGCATCATTCTTGACC
	Rev TCCGAGAACTGCAGTTCTAGG
	T-DNA CTTATTTCAGTAAGAGTGTGGGGTTTTGG
sultr4;1	For AGCTAAGACTGGCCTATCAGG
	Rev CGTTTTGGTGACTCTTGCTC
	T-DNA TGGTTCACGTAGTGGGCCATCG
sultr4;2	For CCAGCAGCATGTATCGGTATC
	Rev ACCGTATATTGGTTGAAGCCC
	T-DNA TGGTTCACGTAGTGGGCCATCG
zir1	For CTGTACTGGAATGACATTTCGG
	Rev AGTCAGCTGTCAGATCCAGG
